# In vivo brain delivery of BBB-enabled iduronate 2-sulfatase in rats

**DOI:** 10.1186/s12987-024-00617-6

**Published:** 2025-01-14

**Authors:** Will J. Costain, Arsalan S. Haqqani, Greg Hussack, Henk van Faassen, Etienne Lessard, Binbing Ling, Eric Brunette, Dao Ly, Hung Fang, Jennyfer Bultinck, Steven Geysens, Gwenda Pynaert, Kathleen Piens, Stefan Ryckaert, Franck Fudalej, Wouter Vervecken, Danica Stanimirovic

**Affiliations:** 1 Human Health Therapeutics Research Centre, National Research Council Canada, Ottawa, ON Canada; 2 Oxyrane, Ghent, Belgium

**Keywords:** Blood brain barrier, MPS II, Iduronate-2-sulfatase, Enzyme replacement therapy, CSF, Brain, Pharmacokinetics

## Abstract

**Background:**

Iduronate-2-sulfatase (IDS) deficiency (MPS II; Hunter syndrome) is a disorder that exhibits peripheral and CNS pathology. The blood brain barrier (BBB) prevents systemic enzyme replacement therapy (ERT) from alleviating CNS pathology. We aimed to enable brain delivery of systemic ERT by using molecular BBB-Trojans targeting endothelial transcytosis receptors. Methods: Single-domain antibody (sdAb)-enzyme fusion protein constructs were prepared in *Yarrowia lipolytica*. sdAb affinity and BBB permeability were characterized using SPR and an in vitro rodent BBB assay, respectively. In vivo pharmacokinetic (PK) analysis was performed in rats. Quantification of fusion protein amounts were performed using LC-MS.

**Results:**

Fusion proteins consisting of IDS and BBB-transmigrating sdAbs, albumin binding sdAbs or human serum albumin (HSA) were evaluated for their in vitro BBB permeability. IGF1R3H5-IDS was selected for in vivo PK analysis in rats. IDS and IGF1R3H5-IDS exhibited very short (< 10 min) serum half-life (t_1/2α_), while constructs containing either HSA or anti-serum albumin sdAbs (R28 or M79) showed 8–11 fold increases in the area under the curve (AUC) in serum. CSF analysis indicated that IGF1R3H5 increased brain exposure by 9 fold (AUC) and constructs containing HSA or R28 exhibited 42–52 fold increases. Quantitation of brain levels confirmed the increased and sustained delivery of IDS to the brain of HSA- and R28-containing constructs. Lastly, analysis of brain fractions demonstrated that the increases in brain tissue were due to parenchymal delivery without fusion protein accumulation in brain vessels.

**Conclusions:**

These results demonstrate the utility of IGF1R-targeting sdAbs to effect brain delivery of lysosomal enzymes, as well as the utility of serum albumin-targeting sdAbs in t_1/2_ extension, to increase brain delivery of rapidly cleared enzymes.

**Supplementary Information:**

The online version contains supplementary material available at 10.1186/s12987-024-00617-6.

## Background

Lysosomal storage diseases (LSDs) are a group of approximately 50 rare inherited metabolic disorders that result from defects in lysosomal function. LSDs are typically the consequence of a deficiency of a single enzyme required for the metabolism of lipids, glycoproteins and mucopolysaccharides. The disease is caused by excessive accumulation of non-processed material (such as heparan sulfate and dermatan sulfate) in cells and tissues, resulting in gross abnormalities in development and mental retardation when the central nervous system (CNS) is affected [1]. At least 75% of all LSDs have a significant CNS pathology [2]. The current standard of care for LSDs is enzyme replacement therapy (ERT), consisting of the intravenous administration of a recombinant form of the affected enzyme. Although ERT has been successful in treating peripheral symptoms by improving enzyme activity in peripheral organs such as liver, kidneys, spleen and heart, it is ineffective in the treatment of CNS pathologies because of the inability of peripherally-administered enzymes to cross the blood-brain barrier (BBB) and consequently reach the CNS [3, 4].

The BBB is formed by specialized endothelial cells of brain microvessels and capillaries joined together by tight junctions that restrict paracellular transport of hydrophilic molecules > 500 Da. Polarized efflux transporters, also present in the BBB, further prevent brain access to many lipophilic synthetic molecules [5]. Systemic delivery of large complex therapeutics, such as enzymes and nanoparticles, across the BBB was not considered feasible until the recent development of novel delivery technologies focused on trans-cellular pathways that harness receptor-mediated transcytosis (RMT) mechanisms [6]. Recent demonstrations of enzyme delivery to the CNS using antibodies targeting the transferrin receptor (TfR) have been reported [7, 8]. However, TfR lacks brain-specific expression and safety concerns emerged during pre-clinical evaluations of TfR antibodies stemming from TfR enrichment in reticulocytes, lungs and neurons [6].

Here, we used single-domain antibodies (sdAbs, VHHs) capable of transmigrating the BBB by RMT (referred to as ‘BBB Trojans’) [9–11], to functionalize CNS delivery of iduronate-2-sulfatase (IDS), an enzyme deficient in patients with mucopolysaccharidosis type II (MPS II; Hunter syndrome). We have previously developed multiple sdAbs capable of BBB transcytosis, with the 1st -generation BBB Trojan FC5 binding transmembrane protein 30 A (TMEM30A [12]), and 2nd -generation BBB Trojans binding insulin-like growth factor 1 receptor (IGF1R [13]). In this work we demonstrate that BBB Trojans, when fused to IDS, enabled BBB transmigration in a rat in vitro BBB model and in rodents. We found a major limitation to CNS delivery was the exceptionally short serum half-life (t_1/2_) of IDS. We addressed this by extending serum t_1/2_ through fusion to either human serum albumin (HSA), or sdAbs that bind serum albumin [14, 15]. Our dual functionalization strategy resulted in dramatic increases in CNS delivery (cerebrospinal fluid and brain) of IDS. Lastly, we used brain fractionation to demonstrate that CNS-enabled IDS was successfully delivered to the brain parenchyma.

## Materials and methods

### Protein design and production

Various recombinant proteins (Supplemental Fig. 1) comprised of IDS alone, or as fusions to sdAbs and/or HSA were designed, expressed and purified. A *Yarrowia lipolytica* yeast strain OXYY5632, co-expressing *Bos taurus* formylglycine generating enzyme [54], was created to produce catalytically active IDS constructs. This strain is glyco-engineered to obtain glycoproteins with high levels of phosphorylated N-glycans [55, 56]. The fermentation and subsequent harvest steps were performed as described previously [55], after which the clarified medium containing the target protein (such as IGF1R3H5-IDS) was subjected to multiple chromatography steps to yield the pure product. The purification protocol consisted of a Ni-immobilized metal-affinity chromatography (IMAC) capturing step to remove the majority of the contaminants, followed by an enzymatic treatment with Jack bean α-mannosidase (JBMan) at pH 4.5 to uncap the shielded mannose-6-phosphate (Man-6-P) and further trim terminal α-linked mannose residues from the protein-linked N-glycans. A second Ni-IMAC step was used to remove JBMan. The final samples were formulated by diafiltration in 20 mM sodium phosphate, 137 mM NaCl, pH 6.2. The same production and purification method was used for all recombinant proteins described in this study. In one case, the IGF1R3H5-IDS-HSA(K573P) construct, hereafter referred to as IGF1R3H5-IDS-HSA, required an extra polishing step to reach the same level of purity as the other constructs.

### Surface plasmon resonance (SPR)

SPR was used to determine the affinities of sdAbs (anti-IGF1R or anti-serum albumin) and IDS fusion constructs for their respective targets, essentially as previously described [15, 41]. SPR analysis was performed at 25 °C on a Biacore T200 or a Biacore 3000 (Cytiva, Vancouver, Canada) using HBS-EP running buffer (10 mM HEPES, pH 7.4, containing 150 mM NaCl, 3 mM EDTA and 0.005% v/v surfactant P20 (polyoxyethylene 20 sorbitan monolaurate); Cytiva), or HBS-EP + running buffer (10 mM HEPES, 150 mM NaCl, 3 mM EDTA, pH 7.4, 0.05% P20; Cytiva), respectively. Human IGF1R (391-GR-050, R&D Systems, Minneapolis, MN) and rhesus IGF1R [41] were immobilized on a CM4 chip at high density (~ 2,500 RUs) in 10 mM acetate buffer, pH 4.0. Variable concentrations of the analytes (sdAbs, IDS fusions) were flowed at 40 µL/min, with contact and dissociations times of 300 and 400 s, respectively. The surface was regenerated in 10 mM glycine, pH 5.5. Similarly, HSA (A3782, MilliporeSigma, Oakville, Canada) or rat serum albumin (RSA, A6414, MilliporeSigma) were immobilized on CM4 sensorchips (~ 1500 RUs of HSA and RSA) in 10 mM acetate buffer, pH 4.5. Variable concentrations of analytes were flowed at 40 µL/min, with contact and dissociations times of 300 and 400 s, respectively. The surface was regenerated in 10 mM glycine, pH 5.5. Reference flow cell subtracted sensorgrams were fit to a 1:1 binding model using BIAevaluation Software v3.2 (Biacore T200) or v4.1 (Biacore 3000).

### In vitro BBB transcytosis model and permeability studies in SV-ARBEC cells

**Fig. 1:**
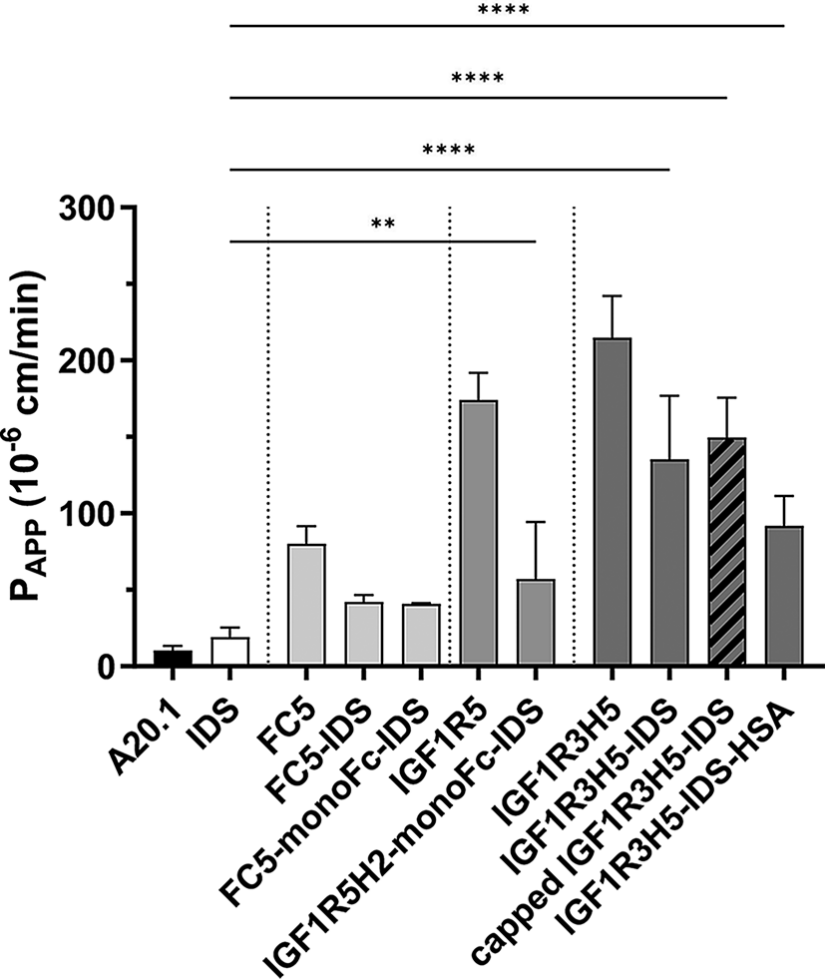
BBB-Trojans enable permeability of IDS in a rat in vitro BBB model. IDS exhibits limited BBB permeability comparable to the negative control A20.1 VHH. Fusions of IDS with BBB-Trojans (FC5, IGF1R5H2 or IGF1R3H5) provide significantly greater BBB permeability relative to IDS and A20.1 VHH. Data are presented as mean ± SD; one-way ANOVA with Šídák’s multiple comparisons test (only significant differences between IDS and IDS fusions are shown), ** *P* < 0.01, **** *P* < 0.0001

SV-ARBECs were seeded at 80,000 cells/membrane on rat-tail collagen-coated 0.83 cm^2^ Falcon cell inserts, 1 μm pore size, in 1 mL SV-ARBEC feeding medium without phenol red. The model characterization is described in detail in Garberg et al. [57]. For cell growth and maintenance prior to the assays, the wells of a 12-well tissue culture plate (i.e., bottom chamber) contained 2 mL of 50:50 (v/v) mixtures of SV-ARBEC medium without phenol red and rat astrocyte-conditioned medium. Pe[sucrose] was determined on inserts dedicated to quality control (QC) assessment for each plating. Transport assays were conducted using the model when Pe[sucrose] was < 0.6 (×10^− 3^) cm/min. The Pe[sucrose] for the QC inserts from the experiments presented in Fig. 1 were 0.40 ± 0.18 × 10^− 3^ cm/min. Transport experiments were performed exactly as described in Haqqani et al. [58] by adding a mixture of the test constructs in equimolar concentrations (2.5 µM) to the top chamber and by collecting 100 µL aliquots (with subsequent replacement with 100 µL of transport buffer) from the bottom chamber at 90 min for simultaneous quantification of all test constructs using the multiplexed selected reaction monitoring (SRM) method. The samples were diluted in transport buffer (TB; 5 mM MgCl_2_, 10 mM HEPES in Hanks’ balanced salt solution (HBSS), pH 7.4) and added (1:1) to the top chamber containing SV-ARBEC media with 5% fetal bovine serum (FBS). For assays where samples were assessed by SRM, the bottom chamber contained TB. For each test construct, negative and positive control constructs were included in the transwell assay. All antibodies were present at the same concentration (2.5 M) in the top well. The A20.1 single domain antibody does not bind to a target on the SV-ARBEC cell surface and does not undergo RMT. A20.1-VHH was present in all inserts along with individual test constructs, was used as a negative control for RMT, and served as an additional QC for barrier integrity. Positive control antibodies (FC5-VHH, IGF1R5-VHH, IGF1R3H5-VHH) were also assessed with A20.1-VHH present in the same transwell inserts. The apparent permeability coefficient P_APP_ was calculated as described previously [59].

### Biodistribution: CSF and brain collection

All animals were purchased from Charles River Laboratories International, Inc. (Wilmington, MA, USA). Animals were housed in groups of three in a 12 h light-dark cycle at a temperature of 24 °C, a relative humidity of 50 ± 5%, and were allowed free access to food and water. All animal procedures were approved by the NRC’s Animal Care Committee and were in compliance with the Canadian Council of Animal Care guidelines. Female Wistar rats aged 8–10 wk (weight range, 135–185 g) were used for sample collection. Cerebrospinal fluid (CSF) and brain were collected to assess the biodistribution of the test articles. The animals were provided analgesia (sustained-release buprenorphine, 1.2 mg/kg) before the first CSF collection, as described previously [58, 60]. CSF was collected and analyzed according to the procedures described in Haqqani et al. [58]. Following CSF collection, the wound was then closed and a blood sample was collected from the tail vein, according to Fluttert et al. [61]. The rat was then returned to its home cage and housed in the recovery room until the next CSF collection. For subsequent CSF and blood collections, the rat was anaesthetized and the sutures removed. The muscles covering the cisterna magna were gently separated and the dura mater exposed. CSF sampling was then performed as described above. Approximately 15–20 µL of CSF can be collected at each time point. For the terminal CSF collection, approximately 50–100 µL of CSF can be collected and blood is collected by heart puncture. Finally, euthanasia is performed by cervical dislocation under deep isoflurane anesthesia.

Following collection of the last CSF sample, rats were sacrificed by cardiac puncture and the brains perfused with 20 mL saline supplemented with 1 EU/mL heparin (Organon, Toronto, Canada) at 2 mL/min and harvested. Brains were sectioned along the midline and the right hemisphere was frozen (-80 °C) and stored for MRM processing.

### Brain homogenization and processing

Prior to MRM analysis the entire right hemisphere was weighed while frozen and the middle third extracted and weighed (typically ~ 0.16 g). The remaining tissue was stored at -80 °C. The brain tissue was then homogenized in 1 mL of ice-cold homogenization buffer (50 mM Tris-HCl, pH 8, 150 mM NaCl, 1.0% sodium deoxycholate (D6750, MilliporeSigma), and 1× protease inhibitor cocktail (P8340, MilliporeSigma)) using a Wheaton Dounce homogenizer (10–12 strokes with a Glas-Col drill (model# 099 C K54) at 60% speed, at 4 °C) until pieces of tissue were no longer detectable. Samples were sonicated (Fisher, Model 300 Sonic Dismembrator) on ice with three 10 s bursts at 30%, and insoluble material was removed by centrifugation (20,000 × *g* for 10 min at 4 °C). The supernatants were then transferred to new tubes on ice. Protein concentrations were then determined using the Bradford method with a standard curve based on bovine serum albumin (BSA Quick Start Standard; BioRad, cat# 500 − 0207). A 5 µL aliquot of the brain extract was diluted 1:5 in 25 mM ammonium bicarbonate (ABC; Sigma, cat# A6141), and a volume corresponding to 20 µg was transferred to a new tube. The 20 µg aliquot was made up to 12.5 µL with 25 mM ABC and 12.5 µL of 10% sodium deoxycholate (DOC; Sigma, cat# D6750) was added to give a concentration of 5% DOC. The samples were then vortexed and briefly centrifuged prior to the addition of 2.5 µL freshly prepared 10× dl-dithiothreitol (DTT; D9163, MilliporeSigma) to provide a concentration of 5 mM DTT. The samples were vortexed and centrifuged briefly and then incubated at 95 °C for 10 min. The samples were then cooled, and briefly centrifuged prior to the addition of 2.75 µL 10× iodoacetamide (I1149, MilliporeSigma) to provide a concentration of 10 mM. The samples were vortexed and centrifuged prior to incubation at room temperature for 30 min in the dark. The samples were then diluted to 125.0 µL with 25 mM ABC. A 2 µL (1 µg) aliquot of trypsin (Promega, cat# V511C) was then added to each sample, which were then mixed gently and briefly centrifuged prior to incubation in a Multitherm Incubator/Chiller unit (model H5000) at 37 °C for 12 h and at 4 °C thereafter. The samples were then stored at -80 °C until MRM analysis was conducted. Prior to MRM analysis, the DOC was precipitated by adding 15 µL AAF buffer (54% acetic acid, 150 mM ammonium acetate, 10% formic acid) to a 115 µL aliquot of the digested sample. The samples were then centrifuged at 50,400 × *g* for 10 min at 4 °C, and 60 µL of the supernatant was transferred to a fresh vial. MRM analysis was performed using 20 µL of the supernatant.

In selected animals, brain homogenates of the left hemisphere were subjected to a vessel depletion protocol to obtain brain parenchyma and brain vessel fractions. The tissues were homogenized as above and sequential filtration through 100 μm and 20 μm nylon Nitex mesh filters (pluriSelect, Leipzig, Germany) was performed to obtain the brain fractions. To verify the efficacy of the vessel depletion procedure, the relative concentrations of markers of parenchyma (glial fibrillary acidic protein, GFAP) and vessels (platelet endothelial cell adhesion molecule, PECAM1) were determined in each sample prior to quantitation of the test articles using SRM as above (Suppl. Figure 2).

### Serum pharmacokinetics analysis

At several post-injection timepoints, blood was collected and serum prepared to determine the serum half-life of the various constructs. Blood samples were taken from the lateral tail vein according to Fluttert et al. [61]. Samples were centrifuged (15 min at 21,100 × g; room temperature) and serum was stored at -80 °C until analysis. Serum and CSF concentration-time profiles were analyzed using WinNonlin software (Version 8.2, Pharsight Corporation, Mountain View, CA, USA).

### Compartmental serum pharmacokinetic analysis

Serum concentration-time data were analyzed using naïve pooled and a two-compartment model with intravenous (i.v.) bolus input, first-order elimination, and macro-rate constants to estimate the following pharmacokinetic parameters: volume of distribution of the central compartment (V_1_) and of the peripheral compartment (V_2_), clearance (CL), inter-compartmental clearance (CL_D_), overall elimination half-life (t_1/2β_) and predicted area under the plasma concentration time curve from time 0 to infinity (AUC). Overall, goodness of fit was based upon the predicted estimate and percent coefficient of variation (% CV) for primary and secondary parameters, as well as inspection of residual plots between observed and predicted concentration-time data.

### Non-compartmental CSF/serum pharmacokinetic analysis

Mean serum and CSF concentration values were used to generate a composite pharmacokinetic profile. A non-compartmental approach consistent with the i.v. route of administration and using the linear/log trapezoidal method was employed to estimate the area under the curve (AUC) of the serum concentration versus time and CSF concentration versus time. For both serum and CSF, the area under concentration versus time curve from the start of dose administration to the last observed quantifiable (AUC_last_) was estimated. Estimation of average concentration ratios for AUC_last_ is reported as (AUC_CSF_/AUC_Serum_) × 100.

### nanoLC/MS/MS

The protein levels of the test constructs in ex vivo samples (serum, CSF and brain) were quantified using targeted nanoflow liquid chromatography tandem mass spectrometry (nanoLC MS/MS). Purified sdAbs, fusion proteins, and body fluid samples containing these proteins were reduced, alkylated, and trypsin digested using previously described protocols [58, 62]. Each protein was first analyzed by nanoLC-MS/MS (nanoAcquity UPLC (Waters, Milford, MA, USA) coupled to LTQ XL ETD MS (ThermoFisher, Waltham, MA, USA)) using data-dependent acquisition to identify all ionizable peptides, and the 3–5 of the most intense fragment ions are chosen. An initial SRM assay was developed to monitor these fragments at attomole amounts of the digest. Fragments showing reproducible intensity ratios at low amounts (~ 100–300 amol; Pearson r^2^ ≥ 0.95) were considered stable and are chosen for the final SRM assay. The locations and identities of the peptides chosen for SRM are shown in Supplemental Fig. 1 and Supplemental Table 1. The blood contamination of CSF samples was evaluated by in-reaction monitoring of rat serum albumin levels using a nanoLC-SRM method as described previously [58]. Measurement of CSF protein concentration was used as a rapid quantitative and nonspecific method for identifying serum contaminated samples. Typical protein concentration of CSF is 0.2–0.4 mg/mL in rat. Protein concentrations > 0.4 mg/mL were considered to be likely contaminated with blood. The serum albumin blood-CSF ratio was determined by multiple SRM analysis of CSF of the corresponding serum sample. Ratios less than 1500-fold were considered contaminated with blood and are excluded from further analyses.

## Results

### Characterization of sdAb-IDS fusion proteins

Recombinant IDS and various fusion proteins containing IDS (Supplemental Fig. 1), harboring high levels of phosphorylated glycans, were produced in the glyco-engineered *Y. lipolytica* strain (OXY5632). The size and identity of the proteins were characterized by SDS-PAGE and western blotting, as well as N-glycan analysis to verify site occupancy and mannose 6-phosphorylation (data not shown). The activity of the IDS domain was determined for all constructs, with specific IDS activity ranging from 15,000 to 30,000 U/mg (data not shown).

Functionality of the monomeric anti-IGF1R and anti-serum albumin sdAbs and various IDS fusion proteins were evaluated by SPR. Table 1 shows that IGF1R3H5, a humanized version of the IGF1R3 VHH, exhibited high affinity (*K*_D_ = 7.5 nM) to human IGF1R. In comparison, the IGFR3H5-IDS construct exhibited ≈ 6-fold increase in *K*_D_ (40–46 nM) for human IGF1R. IDS alone did not bind human IGF1R as expected. The affinity of the IGF1R3H5 domain in the IGF1R3H5-IDS constructs to human IGF1R was not affected by the uncapping process, where the terminal Man-6-P moieties are exposed. The affinity of the IGFR3H5-IDS-HSA (144.7 kDa) construct was *K*_D_ = 77 nM and 84 nM for human and rhesus IGF1R, respectively, and was only slightly weaker than the IGF1R3H5-IDS (78.7 kDa) construct. The reduced affinity of the fusion proteins relative to the monomeric IGF1R VHH may be related to being in an N-terminus fusion configuration. Thus, the IGF1R3H5 fusion construct retains its ability to bind to the target IGF1R.

**Table 1:** SPR-derived kinetics and affinities of VHHs and IDS fusion constructs

Construct	Man6P	Target	k_a_ (1/Ms)	k_d_ (1/s)	K_D_ (M)
IGF1R3H5		h-IGF1R	3.34E + 05	2.51E-03	7.51E-09
IDS		h-IGF1R	n.b.	n.b.	n.b.
IDS		r-IGF1R	n.b.	n.b.	n.b.
IGF1R3H5-IDS	-	h-IGF1R	7.19E + 04	2.89E-03	4.02E-08
IGF1R3H5-IDS	+	h-IGF1R	6.40E + 04	2.95E-03	4.61E-08
IGF1R3H5-IDS		r-IGF1R	n.d.	n.d.	n.d.
IGF1R3H5-IDS-HSA	+	h-IGF1R	3.96E + 04	3.07E-03	7.73E-08
IGF1R3H5-IDS-HSA	+	rh-IGF1R	4.58E + 04	3.86E-03	8.44E-08
M79		HSA	4.52E + 06	0.126	2.78E-08^#^
M79		RSA	1.56E + 07	6.61E-02	1.58E-09^#^
M79		h-IGF1R	n.b.	n.b.	n.b.
R28		HSA	1.14E + 06	1.33E-02	1.17E-08^#^
R28		RSA	2.29E + 06	1.65E-03	7.21E-10^#^
R28		h-IGF1R	n.b.	n.b.	n.b.
IGF1R3H5-IDS-M79		HSA	1.37E + 05	0.265	1.94E-06
IGF1R3H5-IDS-M79		RSA	1.30E + 05	4.36E-02	3.36E-07
IGF1R3H5-IDS-M79		h-IGF1R	n.d.	n.d.	n.d.
IGF1R3H5-IDS-R28		HSA	2.76E + 04	3.29E-02	1.19E-06
IGF1R3H5-IDS-R28		RSA	1.71E + 04	1.51E-03	8.81E-08
IGF1R3H5-IDS-R28		h-IGF1R	n.d.	n.d.	n.d.

IGF1R3H5, humanized VHH targeting insulin like growth factor 1 receptor (IGF1R); IDS, iduronate-2-sulfatase; HSA, human serum albumin (K573P); RSA, rat serum albumin; M79, VHH targeting serum albumin; R28, VHH targeting serum albumin; Man6P, mannose-6-phosphate; h-IGF1R, human IGF1R; r-IGF1R, rhesus IGF1R; n.b., no binding; n.d., not determined

^#^ Determined in van Faassen et al. 2020 [15]

The monomeric anti-serum albumin sdAbs [15] showed low nM affinity toward human and rat serum albumins (Table 1). Similar to the anti-IGF1R sdAbs, the anti-serum albumin sdAbs (R28 and M79) exhibited reduced affinity as C-terminus fusion proteins in the IGF1R3H5-IDS-M79 and IGF1R3H5-IDS-R28 constructs, with *K*_D_s = 1.9 µM and 1.2 µM for HSA, and *K*_D_s = 336 nM and 88 nM for RSA, respectively.

### In vitro analysis of BBB permeability using a rat brain endothelial cell model

An in vitro rat BBB assay was performed to validate the ability of BBB Trojans FC5 [10, 16] and anti-IGF1R sdAbs [11, 17] to enable BBB permeability of IDS, and to identify a lead candidate for subsequent in vivo evaluation. As previously shown, the sdAb FC5 VHH exhibited significantly greater apparent permeability (P_APP_) in the in vitro BBB assay than the non-crossing negative control sdAb A20.1 (a VHH that targets a bacterial toxin and is used here as a negative control for BBB crossing) (Fig. 1) [18]. Furthermore, the permeability of IDS was not statistically different from A20.1, thereby confirming that the enzyme had very low BBB permeability. The fusion constructs comprised of FC5 and IDS exhibited permeability that was significantly greater than A20.1, but substantially reduced in comparison to FC5-VHH and not significantly different from IDS (Fig. 1).

IGF1R-mediated RMT was evaluated using two distinct sdAbs. The P_APP_ of the parental VHHs (IGF1R3 and IGF1R5) was confirmed to be consistent with historical values (IGF1R5, ~ 200; IGF1R3, ~ 250) [11, 19]. A construct comprised of a humanized version of IGF1R5 VHH (IGF1R5H2), monomeric Fc [20], and IDS (IGF1R5H2-monoFc-IDS) also exhibited significantly greater P_APP_ than IDS in the rat BBB assay (Fig. 1). While the P_APP_ of IGF1R5H2-monoFC-IDS was similar to that of FC5, the observed P_APP_ was substantially reduced (33%) in comparison to IGF1R5. A third BBB Trojan (IGF1R3H5, a humanized version of the IGF1R3 VHH) was evaluated and it was found that the P_APP_ of the IGF1R3H5-IDS construct was comparable (66%) to the positive control IGF1R3 (parental llama VHH) and far exceeded that of IDS and A20.1 (9% and 5%, respectively) (Fig. 1). Notably, the P_APP_ of a construct containing HSA(K573P) was comparable to that of IGF1R3H5-IDS (65%). Figure 1 also shows that the permeability of a construct lacking a terminal Man-6-P (capped IGF1R3H5-IDS) was not different from a version with terminal Man-6-P moieties on its glycans. Lastly, the P_APP_ of the IGF1R3H5-based fusion proteins (~ 150) was superior to the FC5 and IGF1R5H2 constructs, was sufficient to justify evaluation in an in vivo transport assay, and therefore selected for subsequent studies.

### In vivo analysis of serum and CSF pharmacokinetics

**Fig. 2:**
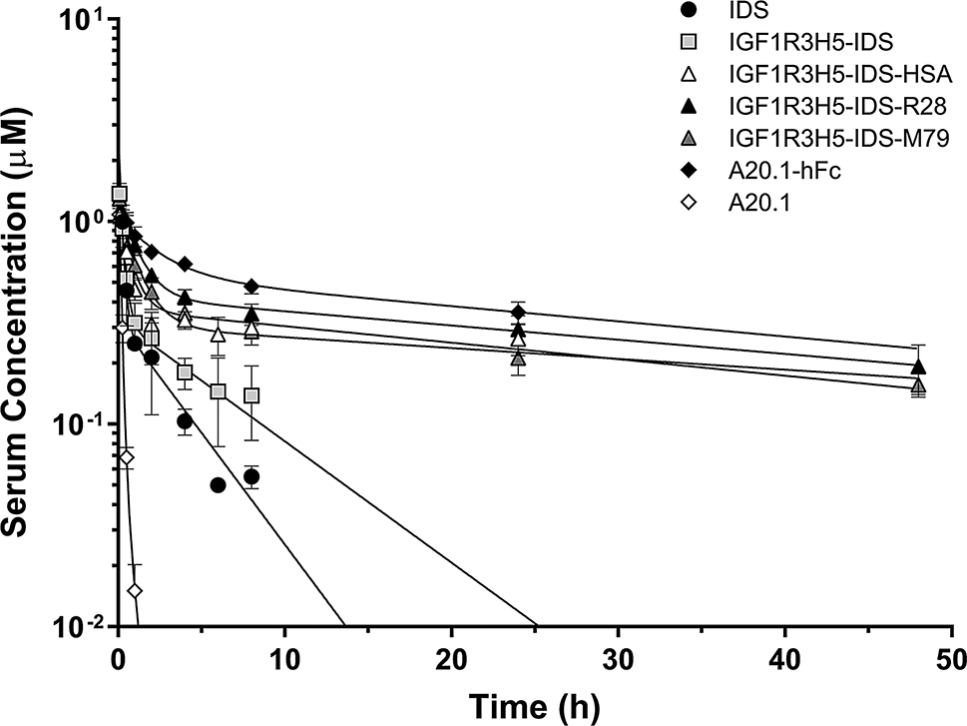
Fusion of IDS with HSA or anti-serum albumin sdAbs (R28, M79) extends the serum half-life of IDS in rats. Serum concentrations of the constructs (observed versus predicted) were determined following i.v. administration at 76 nmol/kg. A20.1 VHH is rapidly eliminated and fusion with human Fc (hFc) dramatically reduced elimination from serum. IDS underwent rapid 2 phase elimination and fusion with HSA, R28 or M79 reduced elimination from serum. Values are shown as mean ± SEM

The constructs IGF1R3H5-IDS and IGF1R3H5-IDS-HSA were selected for in vivo characterization. Two additional constructs comprised of IGF1R3H5-IDS fused to anti-serum albumin sdAbs (R28 and M79) were also evaluated [14]. Figure 2 shows the concentrations of IDS, IGF1R3H5-IDS, IGF1R3H5-IDS-HSA, IGF1R3H5-IDS-R28 and IGF1R3H5-IDS-M79 in rat serum following single bolus i.v. injections of equimolar doses (76 nmol/kg). The data indicate that both IDS and IGF1R3H5-IDS are rapidly cleared from the serum with kinetics (αt_1/2_ and βt_1/2_) that are similar (Table 2), with IGF1R3H5-IDS unexpectedly exhibiting a 57% greater area under the curve (AUC) relative to IDS (Suppl. Figure 3). Similarly, analysis of serum IDS concentrations based on IDS enzymatic activity confirmed that IDS and IGF1R3H5-IDS are rapidly cleared from the serum (Suppl. Figure 4). In comparison, the IGF1R3H5-IDS-HSA construct exhibited a significantly reduced serum clearance (CL) rate (0.07 mL/min/kg) compared to IGF1R3H5-IDS (0.5 mL/min/kg) and IDS (0.7 mL/min/kg). Consequently, IGF1R3H5-IDS-HSA exhibited a much longer serum half-life (αt_1/2_ = 27.8 min, βt_1/2_ = 36.9 h) than IGF1R3H5-IDS and IDS. Correspondingly, the elimination of IGF1R3H5-IDS-HSA was ~ 7- to 11-fold (according to serum AUC values) slower than the constructs lacking HSA (Table 2). The constructs containing the IGF1R- and serum albumin-binding VHHs (IGF1R3H5-IDS-M79 and IGF1R3H5-IDS-R28) exhibited reduced serum clearance and increased serum t_1/2_ values similar to the HSA-containing construct. Unexpectedly, the serum clearance of IGF1R3H5-IDS-M79 and IGF1R3H5-IDS-R28 were less than that observed for IGF1R3H5-IDS-HSA, which is reflected by the serum AUC values for IGF1R3H5-IDS-M79 and IGF1R3H5-IDS-R28 being 1.27- and 1.35-fold greater, respectively, than the AUC for IGF1R3H5-IDS-HSA. Thus, the anti-serum albumin containing constructs exhibited serum PK values that were consistent with the HSA containing construct, indicating that serum half-life extension was realized using two distinct strategies.

**Table 2:** Mean PK parameter estimates (CV%) from 2-compartment analysis of serum concentration-time profiles after intravenous bolus administration of IDS constructs to Wistar rats (n = > 3 per group, 76 nmol/kg)

Parameter	Units	IDS	IGF1R3H5-IDS	IGF1R3H5-IDS-HSA	IGF1R3H5-IDS-R28	IGF1R3H5-IDS-M79
C_max_	nmol/mL	3 (45)	1.8 (8.8)	1.3 (3.3)	1.3 (3.9)	1.2 (6.2)
V	mL/kg	26.2 (45)	44.5 (8.8)	65.1 (3.3)	58.2 (3.9)	65.6 (6.2)
V2	mL/kg	104 (13)	133 (12)	155 (11)	113 (6.2)	172 (11)
CL	mL/min/kg	0.7 (8.9)	0.5 (9.6)	0.07 (34)	0.05 (11)	0.05 (18)
CL2	mL/min/kg	1.4 (22)	1.8 (8)	1.1 (7.8)	0.6 (8.1)	0.7 (12)
AUC	nmol*min/mL	105 (8.9)	165 (9.6)	1212 (34)	1632 (11)	1540 (18)
t_1/2α_	Min	7.5 (29)	11.2 (12)	27.8 (10)	44.4 (11)	46.1 (15)
t_1/2β_	Min	164 (10)	301 (17)	2216 (40)	2574 (14)	3383 (24)

C_max_: Predicted peak concentration. t_1/2α_: Distribution half-life. t_1/2β_: Elimination half-life. CL: total body clearance. CL_2_: Intercompartmental clearance. V_1_: Central compartment volume of distribution. V_2_: Peripheral compartment volume of distribution. AUC: predicted area under the plasma concentration time curve from time 0 to infinity

**Fig. 3:**
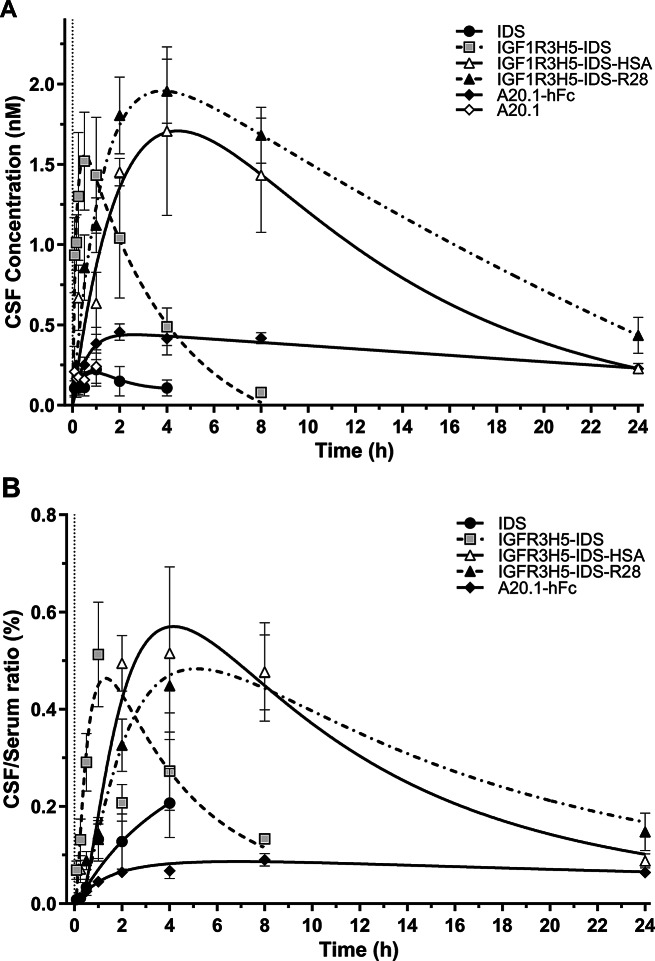
Brain exposure of IDS is enhanced by fusion with IGF1R3H5 and HSA or anti-serum albumin sdAbs (R28, M79) in rats. (**A**) Marginal levels of A20.1 and IDS were detected in CSF following i.v. administration (76 nmol/kg) for up to 1 and 4 h, respectively. Fusion with IGF1R3H5 dramatically increased peak levels in CSF; further fusion with HSA or R28 prolonged the presence of high levels of IDS in CSF. In contrast, levels of A20.1-hFc remained low despite extended presence in serum. (**B**) Analysis of the CSF/Serum showed that the duration of brain exposure reflects the presence in serum, while peak levels are attributable to the inclusion of a IGF1R3H5. Values represent mean +/- SEM

Analysis of test article levels in the CSF was conducted in order to evaluate the ability of IGF1R3H5 to effect BBB transcytosis in vivo. CSF PK is used to measure brain exposure to therapeutics and has been shown to correlate with delivery to brain parenchyma [9, 21]. Figure 3A compares protein concentrations of IDS, IGF1R3H5-IDS, IGF1R3H5-IDS-HSA and IGF1R3H5-IDS-R28 in rat CSF following single bolus i.v. injections of equimolar doses. The peak CSF concentrations of IGF1R3H5-IDS, IGF1R3H5-IDS-HSA and IGF1R3H5-IDS-R28 are similar, which is likely the result of the similar dosing levels employed. This is in stark contrast to the minimal amounts of IDS that were detected in CSF, thus confirming the capability of the IGF1R3H5 antibody to enable in vivo BBB transcytosis of IDS. Analyses of CSF levels of IGF1R3H5-IDS-HSA and IGF1R3H5-IDS-R28 indicate that maximum peak levels were observed 4 h post-administration, with detectable levels present 24 h post-administration (Fig. 3A). In comparison, peak CSF levels of IGF1R3H5-IDS were observed at 30–60 min post-administration and were virtually absent by 8 h post-administration. IGF1R3H5-IDS-HSA levels in CSF at 8 h post-administration were comparable to the peak levels observed for IGF1R3H5-IDS. Plotting the CSF / serum ratio shows that the peak levels in CSF represent ~ 0.5–0.6% of serum levels for the IGF1R3H5-containing constructs (Fig. 3B). Importantly, the IGF1R3H5-IDS-HSA and IGF1R3H5-IDS-R28 constructs exhibited distinctly prolonged CSF PK profiles, resulting in 49- and 61-fold increases, respectively, in the observed AUC compared to IDS (Table 3). Calculation of the AUC ratio (CSF / serum) indicated that the IGR1R3H5-containing constructs exhibited a similar degree of brain exposure, with all constructs being improved by a factor of ~ 3–5 compared to IDS. These data emphasize the dramatic effect of serum half-life prolongation on increasing brain delivery. In contrast, the A20.1-hFc construct exhibited prolonged serum half-life with low CSF levels. As expected, the AUC ratio for A20.1-hFc was comparable to IDS (Table 3). Considering the unexpected serum PK extension observed with the inclusion of IGF1R3H5 (Tables 2 and 3), it appears that IGF1R3H5 and R28 act through an unpredicted mechanism to synergistically extend the serum PK of IDS.

**Table 3:** Mean AUC_last_ of the CSF concentration-time profiles and the ratio over AUC_last_ of the serum concentration-time profiles following intravenous bolus administration of A20.1-hFc or IDS constructs to naïve rats

Parameter	Unit	A20.1-hFc	IDS	IGF1R3H5-IDS	IGF1R3H5-IDS-HSA	IGF1R3H5-IDS-R28
*C*_CSF max_	nmol/L	0.42	0.22	1.56	1.75	1.96
*CSF AUC*_last_	nmol*min/mL	0.50	0.03	0.29	1.47	1.82
*Serum AUC*_last_	nmol*min/mL	717.01	51.45	121.07	466.43	914.63
*CSF AUC*_last_ */ Serum AUC*_last_	%	0.07	0.07	0.24	0.32	0.20

C_CSF max_: Observed maximum concentration in CSF. AUC_last_: area under the plasma concentration time curve from time 0 to the last measured time point

**In vivo analysis of brain levels**.

**Fig. 4:**
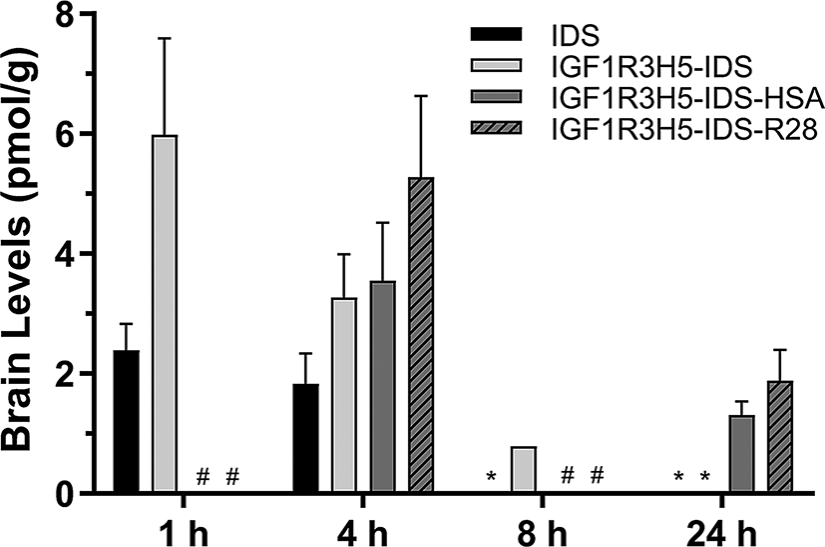
IDS levels observed in whole brain following i.v. administration (76 nmol/kg) in rats. Low (marginal detection) levels of IDS were present in whole brain for up to 4 h post-administration. Fusion with IGF1R3H5 increased peak levels and duration of detection in whole brain. Addition of the half-life extenders (HSA; anti-serum albumin sdAb, R28) resulted in increased levels at 4 h and extension of detection to 24 h post-administration. * not detected; # not determined

**Fig. 5:**
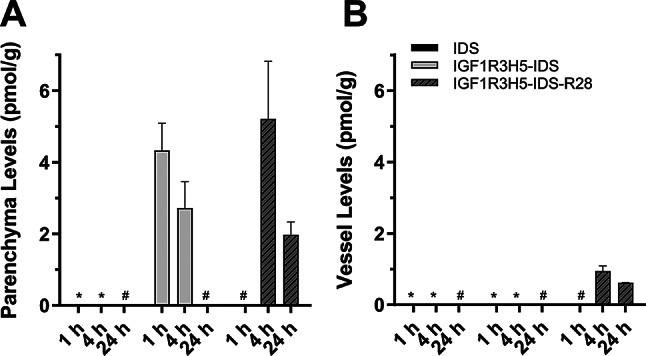
IDS levels observed in brain and vessels following i.v. administration (76 nmol/kg) in rats. (**A**-**B**) Comparison of test article levels over time in brain fractions. IDS was below the detection limits in both brain parenchyma and vessels at all time points. IGF1R3H5-IDS was present in brain parenchyma (**A**) up to 4 h post-administration and IGF1R3H5-IDS-R28 was present in brain parenchyma and vessels (**B**) up to 24 h post-administration. * not detected; # not determined

Whole brain levels of IDS (1, 4 and 8 h), IGF1R3H5-IDS (1, 4, 8 and 24 h), IGF1R3H5-IDS-HSA (4 and 24 h only) and IGF1R3H5-IDS-R28 (4 and 24 h only) were quantified and Fig. 4 shows the test sample levels in rat brain over a 24-h period. Brain levels of IGF1R3H5-IDS were substantially greater than IDS at 1 and 4 h, with IDS no longer detected at 8 h. In comparison, detectable levels of IGF1R3H5-IDS-HSA and IGF1R3H5-IDS-R28 were observed in the brain at 4 and 24 h, indicating an increase in brain delivery that is commensurate with the observed CSF levels. This indicates that overall brain exposure was enhanced by the inclusion of HSA or R28. Figure 5 shows analyses of IDS, IGF1R3H5-IDS and IGF1R3H5-IDS-R28 in brain parenchyma and brain vessels. While IDS was detected in whole brain at 1 and 4 h post-administration (Fig. 4), IDS was below the detection limits in brain parenchyma and vessel samples from the same animals used for whole brain analyses (Fig. 5). In contrast, IGF1R3H5-IDS was detected in whole brain and brain parenchyma at 1 and 4 h. Moreover, IGF1R3H5-IDS was not detected in the brain vessel fraction, while IGF1R3H5-IDS-R28 was detected in whole brain as well as in brain parenchyma and vessels. Importantly, the IGF1R3H5-IDS and IGF1R3H5-IDS-R28 levels in brain parenchyma were much greater than in brain vessels. This indicates that retention of IGF1R3H5-containing constructs in the vessel component is likely to be limited and that delivery to the parenchyma is achieved. An additional experiment was conducted using higher doses (140 nmol/kg) of the test articles. Supplemental Fig. 5 shows IDS and IGF1R3H5-IDS-HSA concentrations in rat brain parenchyma and vessels. The data confirms that IDS is detectable in whole brain at 1 and 4 h, but not at 24 h. The figure also demonstrates IGF1R3H5-IDS-HSA levels were much greater than IDS and the non-BBB crossing controls (A20.1 VHH and A20.1-hFc). Importantly, IDS and A20.1-hFc were not detectable in brain parenchyma at 4 h (levels at 1 and 24 h were not determined), while IDS is present at marginally detectable levels in brain vessels at 4 h. In stark contrast, the majority of IGF1R3H5-IDS-HSA was found in the brain parenchyma and was not observed to be trapped in brain vessels. Furthermore, the low level of brain vessel “trapping” in the in vivo studies is consistent with observations in the in vitro BBB models. To further demonstrate the ability of IGF1R3H5 to enable delivery to brain parenchyma, Supplemental Fig. 6 shows the correlations between test article levels in whole brain and brain fractions. The figure shows that the whole brain levels of the BBB impermeable protein A20.1-hFc exhibited weak correlations with the levels in brain fractions. In contrast, IGF1R3H5-IDS-R28 exhibited a strong correlation between levels in brain and parenchyma, but not between brain and vessels.

## Discussion

Intravenous ERT has been available for Hunter syndrome for over 10 years, and its ability to alleviate somatic symptoms has been documented in numerous clinical trials [22, 23]. However, standard intravenous delivery was found to be of doubtful benefit to patients with mild cognitive impairment, and of no significant benefit to patients with severe cognitive deterioration [24, 25]. The ineffectiveness of first generation ERT in treating the CNS symptoms of lysosomal storage diseases is attributable to the presence of the BBB, which protects the brain from blood components that would interfere with brain function [26]. As an alternative, intrathecal (IT) infusion is a strategy being developed to address the cognitive symptoms of LSDs [3, 25, 27, 28]. While IT administration was shown to reduce glycosaminoglycan concentrations in the CSF of Hunter syndrome patients [25, 29], it remains to be confirmed if cognitive dysfunction can be successfully alleviated using this strategy [30]. Moreover, it is questionable whether or not administration to the CSF can achieve diffuse intraparenchymal penetration in humans [30]. Similarly, clinically implemented medical procedures such as mannitol-induced osmotic BBB disruption [31] and focused ultrasound [32] have yet to show clinical benefits in treating the brain with biotherapeutics. Therefore, the implementation of non-invasive transvascular brain delivery technologies remain critical for enabling successful therapeutic delivery to the CNS.

Antibodies that bind receptors on brain endothelial cells and subsequently undergo RMT have been developed as a means to enable the brain delivery of protein therapeutics [6]. The delivery of IDS by targeting TfR1 for RMT has been developed [8, 33–39]. While strategies targeting TfR1 have been successful in achieving enhanced brain delivery and therapeutic efficacy, TfR1 is broadly distributed throughout the body and lacks BBB specificity [6]. Similarly, antibodies targeting the insulin receptor have been used to deliver enzymes across the BBB [8, 33–39]. Here we have shown that RMT of IDS through the BBB can be achieved by fusion with a sdAb that targets IGF1R (Fig. 1). Previous studies have shown that anti-IGF1R sdAbs are capable of crossing the in vitro and in vivo BBB, as well as delivering physiologically relevant therapeutic levels [40, 41]. In contrast to studies involving fusion of anti-IGF1R sdAbs with Fc or therapeutic peptides (such as galanin or neurotensin), the present study showed that fusion with IDS produced a notable reduction in the affinity of the anti-IGF1R sdAbs toward IGF1R and anti-serum albumin sdAbs toward albumin (Table 1). It should be noted that reduced sdAb affinity is not observed in Fc fusion constructs, indicating that this phenomenon is not simply related to the size of the construct, but rather likely due to some level of steric clash preventing unobstructed binding to the SPR surface and possibly to the target antigens in vivo [42]. Since it is not clear if the reduced SPR-derived affinity is truly physiological, it cannot be cited as the mechanism for the observed reduction in BBB permeability relative to the parent sdAbs (Fig. 1).

When delivered intravenously, IDS is consistently found to be rapidly cleared from the circulation in rodents (t_1/2_ < 2 h) [43, 44], humans and primates (t_1/2_ < 10 h) [45, 46]. While serum t_1/2_ is not representative of tissue t_1/2_, which is reported to be 1–2 days for intravenously administered IDS [47], it does present a limitation in delivery to the brain, which has extremely low exposure to serum proteins [6]. This was clearly shown by Garcia et al. [44], who reported that only a minute proportion of the administered dose was found in the brain and is likely present in the microcirculation rather than the brain parenchyma. In the present study, we confirmed that i.v. administered IDS was rapidly cleared from the circulation (t_1/2α_ < 10 min) in rats, with marginally detectable brain exposure (CSF) and no evidence for the presence of IDS in the brain parenchyma. The rapid clearance of IDS from the blood is the result of uptake by peripheral tissues. It has been reported that > 33% of the injected dose is found in the liver 2 h after IDS administration [44].

The mechanism for cellular uptake of therapeutic enzymes lacking RMT functionality relies on endocytosis mediated by the mannose-6-phosphate receptor (M6PR) or the mannose receptor [4, 47–49]. These receptors are broadly distributed throughout the periphery and lack specificity for neuronal populations. As a consequence, a large amount of the injected enzyme is lost before reaching the brain due to a peripheral ‘sink-effect’ that severely reduces the overall efficacy of the treatment [4, 50–52]. The magnitude of this effect was demonstrated in studies using chemical modification of sulfamidase that disrupts glycan structures [52, 53]. Disruption of Man6-P moieties dramatically decreased endocytosis in fibroblasts, and produced a similarly large prolongation of serum concentrations following i.v. administration. In our study, we compared the in vitro BBB permeability of IDS containing terminal Man-6-P on its glycans (uncapped IDS) with IDS on which the terminal Man-6-P moieties are blocked for interaction with the M6PR (capped IDS). No difference was observed between the capped and uncapped constructs, indicating that Man-6-P-mediated transcytosis did not contribute to BBB permeability (Fig. 1, unpublished observations).

In an attempt to maximize IDS delivery to the parenchyma, we aimed to determine if serum PK could be extended by constructing fusion proteins containing either human albumin or sdAbs that bind serum albumin. As expected, fusion proteins containing either HSA or anti-serum albumin sdAbs exhibited marked increases in serum PK parameters (Fig. 2; Table 2). The consequence of extended serum PK was a dramatic prolongation of brain exposure (Fig. 3, Suppl. Figure 5). Similarly, we showed that delivery to brain parenchyma correlated well with brain exposure and that therapeutically relevant amounts of IDS were present for up to 24 h following a single administration (Fig. 4). Moreover, we were able to confirm that the observed increases in IDS levels within the brain were not due to uptake and retention by the vascular bed (Suppl. Figure 6).

## Conclusions

The study presented here further demonstrates the viability of BBB-permeable IDS-sdAb fusion proteins with extended serum t_1/2_ as a potential therapeutic for Hunter syndrome. This is the first demonstration of an LSD therapeutic with extended serum t_1/2_ that does not require a full Fc domain. The strategy described herein represents a versatile platform technology that is amenable to biomanufacturing and can be redeployed for the numerous other LSDs with CNS pathology.

## Electronic supplementary material

Below is the link to the electronic supplementary material.

Supplementary Material 1

## Data Availability

No datasets were generated or analysed during the current study.

## Abbreviations

ABC: Ammonium bicarbonate
AUC: Under the curve
BBB: Blood-brain barrier
CNS: Central nervous system
CSF: Cerebrospinal fluid
DOC: Sodium deoxycholate
DTT: dl-dithiothreitol
ERT: Enzyme replacement therapy
FBS: Fetal bovine serum
HBSS: Hanks’ balanced salt solution
hFc: Human Fc
HAS: Human serum albumin
iBEC: iPSC-derived brain endothelial cell
IDS: Iduronate-2-sulfatase
IGF1R: Insulin-like growth factor 1 receptor
IMAC: Immobilized metal-affinity chromatography
iPSC: Induced pluripotent stem cell
IT: Intrathecal
JBMan: Jack bean α-mannosidase
LSD: Lysosomal storage disease
Man-6-P: Mannose-6-phosphate
MPS II: Mucopolysaccharidosis type II
P_APP_: Apparent permeability
PK: Pharmacokinetics
RA: Retinoic acid
RMT: Receptor-mediated transcytosis
sdAb: Single-domain antibody
SFTB: Sulfate-free transport buffer
SPR: Surface plasmon resonance
SRM: Selected reaction monitoring
TB: Transport buffer
TEER: Transendothelial electrical resistance
TfR: Transferrin receptor
TMEM30A: Transmembrane protein 30 A
VHH: Variable heavy domain of heavy chain camelid antibody
