# Supplementary material for: In vivo brain delivery of BBB-enabled iduronate 2-sulfatase in rats
Source: Fluids Barriers CNS. 2025 Jan 14;22:7. doi: 10.1186/s12987-024-00617-6 (PMC11734454; doi:10.1186/s12987-024-00617-6)
Supplement: Supplementary file 1 — Supplementary Material 1 [file 12987_2024_617_MOESM1_ESM.docx]

**Supplemental Figure 1.** Schematic of protein constructs evaluated in this study and the locations of peptides monitored by SRM analysis. Iduronate-2-sulfatase (IDS) contains a cleavage sites for processing of an N-terminal propeptide, as well as a internal proteolytic cleavage site for processing into the mature protein comprised of a 42 kDa and 14 kDa polypeptides ^1^. FC5, camelid VHH that binds to TMEM30A ^2^; IGFR3H5, humanized camelid VHH that binds to IGF1R ^3^; HSA, human serum albumin with the K573P mutation; R28, camelid VHH that binds serum albumin ^4,5^; M79, camelid VHH that binds serum albumin ^4,5^. The red underlined regions represent the locations of the peptides used for MS quantitation.

**IDS**

**cleavage site**

**cleavage site**

**FC5**

**IDS**

**cleavage site**

**cleavage site**

**IGFR3H5**

**IDS**

**cleavage site**

**cleavage site**

**IGFR3H5**

**IDS**

**cleavage site**

**cleavage site**

**HSA**

**IGFR3H5**

**IDS**

**cleavage site**

**cleavage site**

**R28**

**IGFR3H5**

**IDS**

**cleavage site**

**cleavage site**

**M79**

**Supplemental Figure 2.** QC of brain fractionation. The vessel depletion method was performed on various brain samples to determine the levels of test articles in brain vessels (samples V1 – V6) and parenchyma (samples P1 – P6). In these experiments, two markers of tissue fractionation were evaluated to confirm the effectiveness of the brain fractionation method. The figure shows the amounts, relative to non-fractionated brain, of [PECAM1](https://www.uniprot.org/uniprotkb/Q08481/entry) (vessel marker) and [GFAP](https://www.uniprot.org/uniprotkb/P03995/entry) (parenchyma marker) in each sample. The figure shows that the tissue markers were depleted or enriched in each sample as expected, thus, the method permitted the verification of the vessel depletion method and identification of samples with insufficient vessel depletion.

**Supplemental Figure 3.** Plot of PK parameters (WinNonlin analysis) presented in Table 2 demonstrating the effect of HSA and anti-serum albumin sdAbs on IDS serum PK.

**Supplemental Figure 4.** Concentration in rat serum following single bolus i.v. administration of IDS or IGF1R3H5-IDS. Serum IDS concentrations were determined by analysis of IDS enzyme activity according to the method of Tolun et al. ^6^. Serum IDS concentrations were similar to those determined using MRM analysis. Similarly, the observed serum half-life (in parentheses, one phase decay model calculated in GraphPad Prism) is similar to the values shown in Table 2.

**Supplemental Figure 5.** *In vivo* PK/BD of IGF1R3H5-IDS following administration to Wistar rats at 140 nmol/kg. Test article levels in serum (**A**) and CSF (**B**) show that, relative to IDS, IGF1R3H5-IDS-HSA has considerably improved persistence in serum and elevated levels in the CSF. While A20.1-hFc exhibits extended presence in the serum relative to A20.1 VHH, it exhibits only marginal brain exposure in CSF. (**C**) Levels of the test articles in whole brain 1, 4 and 24 h post-administration. (**D**) Distribution of test articles in brain fractions at 4 hr post-administration. Marginally detectable levels of IDS, IGFR3H5-IDS-HSA and A20.1-hFc were present in brain vessels, while A20.1 was not detected. While IDS was not detected in brain parenchyma, levels of IGF1R3H5-IDS-HSA were comparable to those observed in whole brain. In contrast, only marginal levels of A20.1-hFc were present in brain parenchyma.

**Supplemental Figure 6.** Correlations between whole brain concentrations and concentrations in brain parenchyma and vessels. Rats received a single bolus i.v. injection of equimolar doses of IGF1R3H5-IDS-R28 or A20.1-hFc. Brain fractions were obtained from the corresponding hemisphere used to determine whole brain concentrations. Test article concentration were determined by MRM. The figure shows that a strong correlation (m = 0.89) between whole brain levels and brain parenchyma was only observed for IGF1R3H5-IDS-R28.

**Supplemental Table 1.** Molecular weights of protein constructs and peptides used for SRM quantitation. Where constructs contained multiple domains, multiple peptides were quantified to verify that the test article was intact.

| **Test Article** | **MW (kDa)** | **SRM Peptide** | **m/z** | **Peptide** |
| --- | --- | --- | --- | --- |
| IDS | 61.6 | IDS Pep1 | 910.9 | QSYFASVSYLDTQVGR |
| FC5-IDS | 76.7 | FC5 Pep1 | 844.9 | ITWGGDNTFYSNSVK |
| IGF1R5H2-monoFc-IDS | 100.1 | IR5H2 Pep1 | 866.9 | GLEWVATIDWGDGGTR |
| IGF1R3H5-IDS | 78.7 | IR3H5 Pep1 | 941.4 | EVQLVESGGGLVQPGGSLR |
| IGF1R3H5-IDS-HSA | 142.7 | HSA Pep1 | 722.3 | VPQVSTPTLVEVSR |
| IGF1R3H5-IDS-R28 | 91.1 | R28 Pep1 | 1113 | EFVAAITNFAGGTTYYADSVK |
| IGF1R3H5-IDS-M79 | 90.7 | M79 Pep1 | 1005.4 | LSCAASGSTFSSSSVGWYR |
| A20.1 | 13.4 | A20.1 Pep1 | 541.3 | EFVAAGSSTGR |
| A20.1-hFc | 39.3 | hFc Pep1 | 937.5 | TTPPVLDSDGSFFLYSK |

**References**

1. Wilson, P. J. *et al.* Hunter syndrome: isolation of an iduronate-2-sulfatase cDNA clone and analysis of patient DNA. *Proc. Natl. Acad. Sci. U. S. A.* **87**, 8531–5 (1990).

2. Stanimirovic, D. B., Sandhu, J. K. & Costain, W. J. Emerging Technologies for Delivery of Biotherapeutics and Gene Therapy Across the Blood-Brain Barrier. *BioDrugs* **32**, 547–559 (2018).

3. Yogi, A. *et al.* Brain Delivery of IGF1R5, a Single-Domain Antibody Targeting Insulin-like Growth Factor-1 Receptor. *Pharmaceutics* **14**, (2022).

4. Henry, K. A., Tanha, J. & Hussack, G. Identification of cross-reactive single-domain antibodies against serum albumin using next-generation DNA sequencing. *Protein Eng. Des. Sel.* **28**, 379–83 (2015).

5. van Faassen, H. *et al.* Serum albumin-binding VH Hs with variable pH sensitivities enable tailored half-life extension of biologics. *FASEB J.* **34**, 8155–8171 (2020).

6. Tolun, A. A. *et al.* A novel fluorometric enzyme analysis method for Hunter syndrome using dried blood spots. *Mol. Genet. Metab.* **105**, 519–21 (2012).
